# Compensation of Wild Plants Weakens the Effects of Crop-Wild Gene Flow on Wild Rice Populations

**DOI:** 10.3389/fpls.2021.681008

**Published:** 2021-07-13

**Authors:** Dongxin Ouyang, Shanshan Dong, Manqiu Xiao, Jianling You, Yao Zhao, Yuguo Wang, Wenju Zhang, Ji Yang, Zhiping Song

**Affiliations:** ^1^The Ministry of Education Key Laboratory for Biodiversity Science and Ecological Engineering, Institute of Biodiversity Science, Institute of Botany, Tibet University-Fudan University Joint Laboratory for Biodiversity and Global Change, Fudan University, Shanghai, China; ^2^Nanjing Institute of Environmental Sciences of the Ministry of Ecology and Environment, Nanjing, China

**Keywords:** compensation, crop-wild gene flow, fitness, hybrid vigour, tolerance, transgene

## Abstract

Crop-wild gene flow may alter the fitness of the recipient i.e., crop-wild hybrids, then potentially impact wild populations, especially for the gene flow carrying selective advantageous crop alleles, such as transgenes conferring insect resistance. Given the continuous crop-wild gene flow since crop domestication and the occasionally stressful environments, the extant wild populations of most crops are still “wild.” One interpretation for this phenomenon is that wild populations have the mechanism buffered for the effects of crop alleles. However, solid evidence for this has been scarce. We used wild rice (*Oryza rufipogon*) and transgenic (*Bt/CpTI)* rice (*O. sativa*) as a crop-wild gene flow model and established cultivated, wild, and F7 hybrid rice populations under four levels of insect (*Chilo suppressalis*) pressure. Then, we measured the trait performance of the plants and estimated fitness to test the compensatory response of relatively high fitness compared to the level of insect damage. The performance of all plants varied with the insect pressure level; wild plants had higher insect-tolerance that was expressed as over- or equal-compensatory responses to insect damage, whereas crop and hybrids exhibited under-compensatory responses. The higher compensation resulted in a better performance of wild rice under insect pressure where transgenes conferring insect resistance had a somewhat beneficial effect. Remarkable hybrid vigour and the benefit effect of transgenes increased the fitness of hybrids together, but this joint effect was weakened by the compensation of wild plants. These results suggest that compensation to environmental stress may reduce the potential impacts of crop alleles on wild plants, thereby it is a mechanism maintaining the “wild” characteristics of wild populations under the scenario of continuous crop-wild gene flow.

## Introduction

Crop-wild gene flow has occurred continuously since the domestication of crops, allowing crop alleles to introgress into wild populations (Ellstrand et al., 2013). The introgressive crop alleles are presumably highly deleterious in the wild populations or enhance the fitness of the resulting hybrid (Ellstrand, 2003; Stewart et al., 2003; Hovick et al., 2012; Ellstrand et al., 2013; Campbell et al., 2016), therefore crop-wild gene flow may lead to ecological and evolutionary consequences for the recipient wild populations (Burke and Rieseberg, 2003; Lu et al., 2009; Merotto et al., 2016; Mitchell et al., 2019). Previous studies indicate that crop alleles transferred through crop-wild gene flow can persist in the wild populations for a long time (Whitton et al., 1997; Snow et al., 2009). However, the persistent crop alleles do not always alter the performance of recipient wild populations, consequently the character of extant wild populations of most crops are still “wild.” For example, common wild rice *Oryza rufipogon* Griff., the ancestor of the Asian cultivated rice *O. sativa*, has been exposed to crop-wild gene flow since rice was domesticated ca. 9,000 years ago (Fuller et al., 2010). Although a considerable proportion of the current *O. rufipogon* populations harbours domestication genes transferred from cultivated rice by gene flow (for instance, *sh*4 and *PROG*1 that are respectively responsible for two of the most critical domestication traits for rice: non-shattering grains and erect growth) (Wang et al., 2017), the wild rice populations still retain “wild” characteristics: typically displaying the traits of long awns, severe seed shattering, proanthocyanidin-pigmented pericarps, open panicles with few secondary branches bearing relatively few grains, and a non-erect to prostrate/creeping growth habit (Zheng et al., 2019). To explain this paradox, it is hypothesised that wild rice populations have evolved mechanisms to compensate the negative influence of crop gene flow on the seed bank and perennial habit of the wild population (Wang et al., 2017). But this hypothesis has never been examined.

It is well-known that whether crop-wild gene flow leads to potential consequences for wild plants is primarily determined by the fitness performance of the recipient plants, i.e., crop-wild hybrids. Therefore, elucidating the factors influencing crop-wild hybrid fitness can provide insights to the mechanisms influencing the effects of crop-wild gene flow on wild populations. This elucidation is also critical for the concern on the ecological risk of transgene escaping as this transgene crop-wild gene flow may give the recipient plants some ecologically important characteristics (Mercer et al., 2007). In the last two decades, to forecast the potential risks of the commercialisation of transgenic crops, a growing number of studies have focused on the fitness of crop-wild/weed hybrids and the effect of selective advantage cultivated genes. These studies together provide us an opportunity to analyze the factors influencing crop-wild hybrid fitness. A rough literature search included a total of 328 observations reported in 39 independent studies (1998~2020) focusing on crop-wild/weed hybrid performance. The search showed that more than 63% of hybrids performed better or similar to wild plants regardless of the presence of transgenes and growing conditions, while only ca. 39% transgenic hybrids outperformed wild plants, although the benefit effect of transgenes on hybrid fitness is remarked upon under the target pressure conditions (Supplementary Figure 1). These results generally demonstrate that the performance of crop-wild hybrids is affected by hybrid vigour, crop alleles (transgenes), the growing environment, and their interactions. They also suggest that crop-wild gene flow does not always alter the performance of wild populations even if it transferred selective traits or alleles into wild populations, hinting that there is unknown mechanism(s) weakening the effect of crop-wild gene flow (Supplementary Figure 1). In addition, this research never consider the effect of compensation on the fitness of crop-wild hybrid and wild plants.

The plants have the ability to tolerate stressful conditions and damage (such as insect or/and disease injection, drought or flooding, etc.) by compensating for the loss of biomass and thereby recovering form damage either partly or completely (McNaughton, 1983; Strauss and Agrawal, 1999). The degree of tolerance of a plant is usually referred to as compensation, such as under-, equal-, or over-compensation which is related to the intensity of the pressure (Supplementary Figure 1; Strauss and Agrawal, 1999). Compensation can be expressed as the relative fitness by comparing the performance of damaged plants with undamaged counterparts. Crops are usually more sensitive to biotic or abiotic pressure than wild plants, showing different compensatory responses between crops and wild relatives under the same conditions (Warschefsky et al., 2014; Seiler et al., 2017). While the resistance conferred by transgenes may reduce the preference of herbivores (Agrawal, 2000). Xia et al. (2016) found that *Bt* transgene crop-weedy rice F1 hybrids produced 11.7–109.9% more seeds than corresponding non-transgene hybrids under high-insert pressure. Such a pattern was also detected in *Bt* transgene crop-wild rice F1~F2 hybrids by Li et al. (2016). For wild plants, the negative impacts of environmental stress can be fully compensated by an equal-compensation only, their fitness can even be enhanced if an over-compensation happens. Huhta et al. (2000) reported that over-compensation induced by both 10 and 50% biomass removals increased the number of fruits 1.7- and 2.3-flod in *Gentianella campestris*, respectively. Considering hybrid and wild plants under the same stressful conditions, hybrid weakness relative to its wild parents would be expected if the lower compensation of crops was inherited by the crop-wild hybrids and the negative effect of environmental stress is not overcome by the resistance of novel crop alleles. In this scenario, those hybrids would be eliminated by the competition from the wild plants, and then the consequences of crop-wild gene flow should be negligible (Li et al., 2016). Therefore, compensation may play an important role on weakening the impacts of crop-wild gene flow on wild populations. However, neither the effects of compensation solely nor the effects of the interaction between compensation and transgenes on plant performance has been tested soundly.

To evaluate the effect of compensation, as well as that of hybrid vigour, crop alleles and environmental conditions, on the fitness of crop-wild hybrids, we used the insect-resistant transgene (*Bt/CpTI* gene) as a proxy for crop alleles that may influence the fitness performance of crop-wild hybrids (Snow et al., 2009), and established artificial populations of wild, cultivated (with or without the *Bt/CpTI* gene) and hybrid rice (transgenic or non-transgenic). We produced the crop-wild hybrid populations to F7 generation (Baack et al., 2008), while slight hybrid vigour is already detected in F1 - F4 generation of crop-wild/weedy rice hybrids (e.g., Song et al., 2004; Yang et al., 2012; Li et al., 2016). We grew them in an agricultural field in Shanghai (China) under four levels of infestation of the striped rice borer *Chilo suppressalis* (Walker) as follows: no infestation, and low, medium and high infestation levels. We investigated insect damage and insect population size in plant populations and quantified the plant population's growth and reproductive performance under different insect infestation levels. We then compared the lifetime fitness of each plant between pressure levels and the relative fitness between transgenic and non-transgenic hybrids, between hybrids and wild plants, and between cultivated and wild plants under the same conditions to test the hypotheses: (1) there is different tolerance to insect damages between rice types and it will influence the plant's fitness performance; (2) the final fitness of hybrid is determined by the interaction of the effects of transgenes, hybrid vigour, compensation, and growing environmental conditions. Especially, we want to answer the following questions: (1) Are there significant effects of transgenes on a hybrid's fitness performance under insect pressure gradient? (2) Is there significant hybrid vigour, if so, will it add to the transgene effects then enhance hybrid's performance? (3) Do wild plants display higher compensation to insect damage? Based on the answers of these questions, we can infer the interactive effects of the above-mentioned factors on a hybrid's fitness, then the mechanism influencing the effects of the crop-wild gene.

## Materials and Methods

### Plant Material

Five types of rice were used: common wild rice (coded as W), the transgenic rice line Kefeng8 (coded as KF8) bred beyond the seventh generation from the inbred traditional Minghui86 variety, Minghui86 (coded as MH86), and the transgene-positive hybrid (coded as TP) and transgene-negative hybrid (coded as TN) of W and KF8. KF8 and MH86 were provided by the Fujian Province Key Laboratory of Genetic Engineering for Agriculture, Fujian Academy of Agricultural Sciences, China. KF8 harbours the double-gene *Bt/CpTI* which was obtained by *Agrobacterium*-mediated transformation of *Bt Cry1A(c)* (*Bacillus thuringiensis*) and *CpTI* (cowpea trypsin inhibitor) into the borer-sensitive rice line MH86. The insect-resistant transgenic line was developed to control lepidopteran pests, such as the rice stem borer [*Chilo suppressalis* (Walker), etc.] and leaf roller (*Cnaphalocrocis medinalis*). Common wild rice was collected from natural populations in Jiangxi Province, Hunan Province, and Guangdong Province, China. We used KF8 as a paternal parent and 10 accessions of wild rice from the abovementioned populations as maternal parents to breed crop-wild hybrids. True F1 hybrids were identified by a specific *Bt* toxin protein ELISA reagent strip (Beijing Anjiai Biochemistry Technology Development Co. LTD). Then the self-bred advanced hybrid progenies (F6) were screened by ELISA reagent strip to divide them into transgene-positive and transgene-negative populations. At the same time, the 10 wild accessions were also planted to produce seeds with open pollination. All seeds of F7 plants and wild accessions were respectively mixed to form the TP, TN and W populations to avoid the genetic effects of different families. Finally, F7 hybrids (TP and TN), wild rice (W), KF8, and MH86 were used for the determination of fitness.

### Insect Collection

*C. suppressalis* was the target insect of *Bt/CpTI* transgene. This species is one of the most serious pests in rice production (Khan et al., 1991), has a life cycle of 45 to 65 days and may produce 2–5 generations a year in China. We collected the egg masses of *C. suppressalis* directly from the rice fields in Jiangxi Province, China, hatched the eggs under greenhouse conditions and fed the larvae on MH86 seedlings in a 2 × 2 × 2 m nylon net outdoors until the 3rd instar life stage (~8 mm in length), and then used the instars as the experimental insect population.

### Field Experiment

Field trials were conducted at the agricultural field of Fudan University from May to November 2017. All rice seeds were treated for 3 days at 45°C to break dormancy and were then germinated at 30/25°C (day/night, 12/12 h). The germinated seeds were sown in a seedbed outdoors. Thirty-day-old rice seedlings were transplanted into the experimental plots (May 30, 2017). Each plot had an area of 2 × 2 m and contained 81 individual plants of one rice type with a spacing of 20 cm between each plant. Each plot was covered with a 2 × 2 × 2 m nylon mesh to isolate the pests. There were three replicates of each rice population under one level of insect pressure, and four levels of insect pressure were used; thus, a total of 4 × 5 × 3 = 60 rice populations (plots) were established. All the plots grew in a complete randomised design with a 60-cm spacing between plots and with the same water-fertiliser management. To avoid seed loss by shattering, the panicles of each W, TP, and TN plant were enclosed in a nylon bag 15 days after flowering.

According to the definition of the classification index of five occurrence degrees in the Chinese National Standards “Rules of investigation and forecast for the Asiatic rice striped borer (*C. suppressalis*)” (GB/T15792-2009), we set up four levels of insect pressure: (1) zero, no insect; (2) low, 6 larvae/m^2^; (3) moderate, 12 larvae/m^2^; and (4) high, 24 larvae/m^2^ per plot. We used 3rd instar larvae to infest rice plants to ensure the initial insert pressure to be similarly efficient in different populations (Qin and Xie, 1991). The infestation was carried out at the jointing stage of rice (July 30, 60 days after transplantation) when the 3rd instar larvae often begin to infest rice stems and cause yellowing, withering, or death of rice tillers under natural conditions. We put the active larvae of the same length into an Eppendorf tube (EP tube) carefully with a brush, quickly laid the tube at the flag leaf axil of the main rice tiller, and then opened the tube to allow the larvae to climb out and drill holes in the stem. A total of 12 plants were infested by *C. suppressalis* in each plant population, and the positions of infested plants were consistent in all plots (Supplementary Figure 2), while the insect numbers varied according to the level of insect pressure: 2 larvae were used per tube for low pressure; 4 larvae per tube for medium pressure; and 8 larvae per tube for high pressure.

### Population Investigation and Trait Measurement

We recorded the numbers of blasted tillers caused by *C. suppressalis* at the first-flowering, full-blossom and ripening stages in each rice population. These time points corresponded to the 5th instar larvae stage, the emergence stage and the 5th instar larvae stage of the next generation of *C. suppressalis*, respectively. Because the number of blasted tillers varied among the three investigations and reach the maximum value at the third survey, we used the 3rd dataset to characterise the global degree of insect damage in every population. We split every tiller of the harvested rice plants carefully to count the number of *C. suppressalis* larvae inside. The population density of *C. suppressalis* was calculated as the sum of the harvested larvae/the number of harvested plants.

After the seeds had filled, to avoid the marginal effects, four subplots were established in the centre of each plot for plant sampling (Supplementary Figure 2). Each subplot contained four plants that were sampled individually, as a result, a total of 16 plants were characterised in a plot. We measured the height and the number of tillers and panicles of each sampled plant and then harvested the aboveground materials to measure the following: number of filled seeds per panicle, number of filled seeds per plant, seed weight per plant, 100-seed weight, aboveground biomass, effective tillers, and biomass investment in growth. The details of the measurements are listed in Supplementary Table 1.

### Data Analysis

We first used two-way ANOVA to examine the effects of rice types (W, TP, TN, KF8, MH86), insect pressure levels (zero, low, moderate, and high) and their interactions on insect damage and fitness-related traits, Duncan's Multiple Range Test was then used to analyze the differences in fitness-related traits of the same population among four insect pressures and among five populations under the same insect pressure. A chi-square test was used to determine whether the *C. suppressalis* population increased with the number of initially released insects.

Secondly, the fitness-related traits were grouped based on two main life-history stages: the growth and reproductive stages, then the composite fitness was estimated across the entire life history (Burke et al., 1998; Song et al., 2004; Zhang et al., 2018) (Supplementary Table 1). Particularly, considering the importance of clonal reproduction via tillers and stolons in maintaining the perennial habit of the wild parent *O. rufipogon*, we further grouped the fitness components according to traits associated with clonal or sexual reproduction. The relative performance of each trait was averaged in terms of the life stages and the composite fitness across the entire lifetime were obtained by averaging the fitness estimates of growth, clonal, and sexual reproduction. We also estimated the relative fitness as follows: (1) the fitness of hybrids (TP and TN) relative to the wild parent (W) *F*_*TP*/*W*_ and *F*_*TN*/*W*_ under the same insect treatment; (2) the fitness of transgenic (TP or KF8) relative to non-transgenic (TN or MH86) rice types *F*_*TP*/*TN*_ and *F*_*KF*/*MH*86_ under the same insect treatment; (3) the relative fitness of wild rice (W) and hybrids (TP and TN) under insect pressure compared to that under no insect pressure, *F*_*W*−*n*_, *F*_*TN*−*n*_, and *F*_*TP*−*n*_ [the letter *n* indicates the insect pressure level: low (l), medium (m), or high (h)] under low, medium, and high insect pressures to the corresponding values under no insect pressure, respectively (Mercer et al., 2007). One-way ANOVA was used to examine the effects of insect pressure levels on fitness at different life-history stages and on composite fitness. A *t*-test was used to examine whether *F*_*TN*/*W*_, *F*_*TP*/*W*_, and *F*_*TP*/*TN*_ was >1.00 or <1.00 under each insect pressure level to reveal the effects of hybrid vigour and transgenes on fitness. Two-way ANOVA was further used to examine the effects of rice types and insect pressure levels on fitness (*F*_*W*−*n*_, *F*_*TN*−*n*_, and *F*_*TP*−*n*_). The differences in relative fitness of plant (*F*_*W*−*n*_, *F*_*TN*−*n*_, and *F*_*TP*−*n*_) were examined using Duncan's Multiple Range Test to quantify the degree of compensation (tolerance) following Belsky (1986). The differences between *F*_*TP*/*W*_ and *F*_*TN*/*W*_ or *F*_*TP*/*TN*_ and *F*_*KF*/*MH*86_ were detected using chi-square (χ^2^) test. All statistical analyses were performed using the software package IBM SPSS ver. 19.0 for Windows (SPSS Inc., IBM Company Chicago, IL, USA, 2010). Where necessary, the response variables were transformed (square, fourth root or log, depending on the variable) to meet the assumptions of the analyses.

## Results

### Insect Density and Damage

**Figure 1:**
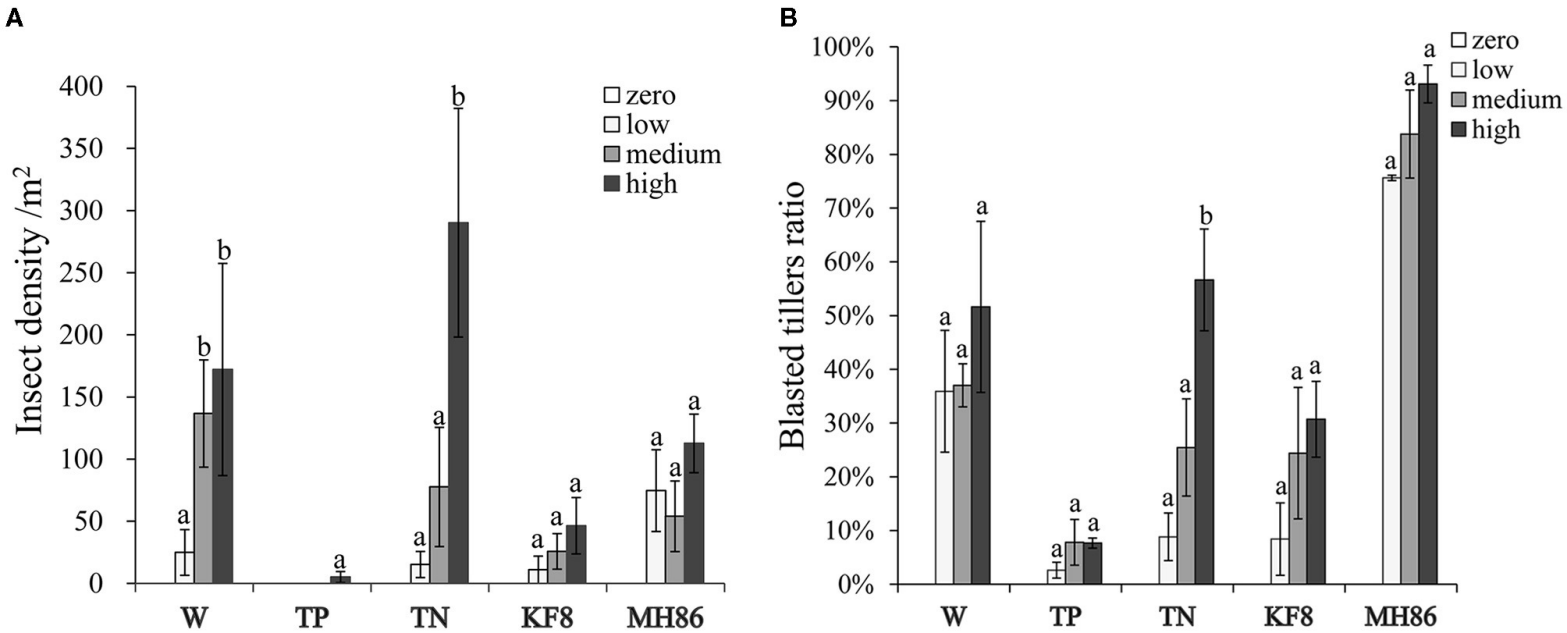
The final insect density **(A)** and percentage of blasted tillers **(B)** in populations of wild rice (W), transgenic and non-transgenic hybrids (TP, TN), and transgenic and non-transgenic rice lines (KF8, MH86) under different insect infestation intensities. Bars represent the standard error. Different letters denote their significant differences (*P* < 0.05) using the Duncan's multiple range test.

The results of two-way ANOVA showed that the *C. suppressalis* density and damage significantly differed between plant populations and between insect pressure treatments (Table 1). Few insects survived in the transgenic populations TP and KF8 (except for TP at high pressure), while a large number of *C. suppressalis* occurred in the populations MH86, W, and TN (Figure 1A; Supplementary Table 2), in which the final insect density increased with the pressure level, but the rates of increase did not correspond with the initial gradient (χ^2^, *p* > 0.05). Except for the TP population, the degree of *C. suppressalis* damage in plant populations increased with the insect pressure level (Figure 1B). The transgenic populations TP and KF8 suffered lower insect damage than the non-transgenic populations. The degree of insect damage in the TN population was similar to that in the W population.

**Table 1:** *F*-values generated from two-way ANOVA testing the effects of insect pressure, rice type, and their interaction on insect damage and final insect density and fitness-related traits.

**Source**	***df*^a^**	**Blasted tiller ratio**	**Final insect density**	***df***	**No. of tillers**	**No. of panicles**	**Plant height (cm)**	**Aboveground biomass (g)**	**No. of filled seeds**	**Weight of filled seeds (g)**	**100-seed weight (g)**	**Growth investment (g)**	**Ratio of effective tillers per plant**	**No. of filled seeds per panicle**
Rice type	4^b^	39.505^**^	5.485^**^	2^c^	1.35	3.421^*^	67.568^**^	2.634	45.482^**^	38.691^**^	36.739^**^	0.554	26.201^**^	30.737^**^
Pressure	2^d^	9.81^**^	7.91^**^	3	2.469	6.126^**^	4.022^**^	10.653^**^	5.746^**^	7.935^**^	5.127^**^	9.345^**^	22.126^**^	1.52
Rice type × Pressure	8	1.099	2.31^*^	6	5.617^**^	3.391^**^	1.163	3.704^**^	4.010^**^	5.121^**^	1.351	3.158^**^	3.35^**^	1.431

*To meet the normality of the residuals from the two-way ANOVA, data on blasted tillers were fourth root transformed; data on the number of filled seeds was square root transformed*.

a *df, degrees of freedom*.

b *Five rice types were compared*.

c *Three rice types, W, TP, and TN were compared*.

d *Low- medium- and high-insect pressures were compared with no insect pressure conditions*.

* *P < 0.05*;

** *P < 0.01*.

### Performance of Fitness-Related Traits

The two-way ANOVA revealed that the fitness-related traits were different between populations of each rice type under different insect treatments and between different plant populations under the same level of insect pressure (Table 1). Under no pressure, nearly all the traits of the hybrids (TP and TN) were similar, they both performed better than the wild parent (W) (Table 2), with 205 and 275% increases in the numbers of filled seeds and 25 and 31% increases in the aboveground biomass. No significant differences were found in the traits between KF8 and MH86 or between TP and TN (Table 2).

**Table 2:** Average values and standard errors (±) of the fitness-related traits of transgene-positive (TP), and transgene-negative (TN) crop-wild hybrid progenies, wild rice (W), transgene-positive cultivated rice (KF8), and transgene-negative cultivated rice (MH86) under four insect pressures.

**Life-history stage**	**Trait**	**Insect pressure**	**Rice type**				
			**W**	**TP**	**TN**	**MH86**	**KF8**
Growth	Plant height (cm)	Zero	153.60 ± 5.72^Ab^	201.00 ± 2.45^Bc^	192.00 ± 5.91^Bc^	108.21 ± 0.98^Ba^	101.37 ± 1.65^Aa^
		Low	165.28 ± 5.44^Ab^	198.51 ± 3.38^Bd^	182.71 ± 4.46^ABc^	107.94 ± 0.92^Ba^	101.39 ± 1.35^Aa^
		Medium	153.72 ± 5.12^Ab^	191.85 ± 4.23^ABc^	186.78 ± 5.75^ABc^	101.77 ± 1.34^Aa^	106.47 ± 1.27^Ba^
		High	151.36 ± 5.17^Ab^	187.43 ± 3.49^Ad^	172.12 ± 5.15^Ac^	102.35 ± 1.45^Aa^	100.25 ± 1.33^Aa^
	Aboveground biomass (g)	Zero	34.14 ± 3.40^ABb^	44.54 ± 2.8^Bbc^	51.95 ± 3.68^Bc^	24.22 ± 1.04^Cba^	24.42 ± 1.56^Aa^
		Low	38.96 ± 3.60^Bc^	35.73 ± 2.32^Ac^	30.62 ± 2.14^Abc^	23.30 ± 1.61^BCa^	25.86 ± 1.48^Aab^
		Medium	31.61 ± 3.93^ABb^	32.70 ± 2.52^Ab^	31.90 ± 2.78^Ab^	17.73 ± 1.29^Aa^	27.69 ± 1.48^Ab^
		High	26.90 ± 2.33^Ab^	35.39 ± 2.73^Ac^	33.17 ± 2.02^Ac^	19.98 ± 1.06^ABa^	27.18 ± 1.44^Ab^
	Growth investment (g)	Zero	32.44 ± 3.30^ABb^	38.95 ± 2.36^Bc^	45.30 ± 3.39^Bcd^	17.54 ± 0.86^Ca^	16.82 ± 1.16^Aa^
		Low	36.44 ± 3.45^Bc^	31.34 ± 2.09^Abc^	27.22 ± 1.93^Ab^	16.25 ± 1.18^BCa^	18.35 ± 1.08^Aa^
		Medium	30.00 ± 3.77^ABb^	27.46 ± 1.98^Ab^	28.87 ± 2.60^Ab^	12.14 ± 0.93^Aa^	16.69 ± 0.89^Aa^
		High	25.21 ± 2.13^Ac^	31.28 ± 2.47^Ad^	30.03 ± 1.86^Ad^	13.99 ± 0.84^ABa^	18.93 ± 1.00^Ab^
Clone reproduction	No. of tillers	Zero	5.33 ± 0.36^Aa^	6.51 ± 0.37^Ab^	7.57 ± 0.42^Cc^	5.96 ± 0.36^Bab^	7.02 ± 0.47^Bbc^
		Low	7.20 ± 0.53^Bb^	5.68 ± 0.35^Aa^	4.98 ± 0.32^Aa^	5.20 ± 0.34^ABa^	6.00 ± 0.32^Aa^
		Medium	6.58 ± 0.52^ABb^	5.51 ± 0.30^ABa^	6.19 ± 0.30^Bab^	4.75 ± 0.29^Aa^	6.36 ± 0.35^ABb^
		High	6.36 ± 0.46^ABb^	6.56 ± 0.60^Ab^	7.48 ± 0.57^Cb^	5.13 ± 0.27^ABa^	7.06 ± 0.37^Bb^
Sexual reproduction	No. of filled seeds per panicle	Zero	19.13 ± 3.51^Aa^	40.06 ± 3.69^Ab^	44.59 ± 3.93^Bbc^	54.59 ± 3.83^Ac^	52.09 ± 4.62^Abc^
		Low	24.10 ± 3.42^Aa^	40.31 ± 3.69^Ab^	33.64 ± 3.32^Aab^	72.27 ± 12.75^ABc^	60.51 ± 6.01^Ac^
		Medium	16.56 ± 2.71^Aa^	45.68 ± 5.68^Ac^	34.52 ± 4.74^Ab^	53.76 ± 4.46^Ac^	83.95 ± 5.90^Bd^
		High	18.91 ± 3.23^Aa^	35.01 ± 4.19^Ab^	30.19 ± 3.79^Ab^	65.17 ± 7.91^Ac^	59.13 ± 4.44^Ac^
	No. of panicles	Zero	5.02 ± 0.35^Aa^	6.24 ± 0.36^Abc^	7.41 ± 0.42^Ccd^	5.96 ± 0.36^Bbc^	6.85 ± 0.47^Ac^
		Low	5.53 ± 0.55^Aab^	5.43 ± 0.94^Aab^	4.49 ± 0.29^Aa^	5.03 ± 0.31^Aab^	5.85 ± 0.33^Ab^
		Medium	4.74 ± 0.49^Aa^	5.07 ± 0.30^Aa^	4.71 ± 0.31^Aa^	4.44 ± 0.25^Aa^	6.17 ± 0.34^Ab^
		High	4.51 ± 0.49^Aa^	5.70 ± 0.55^Ab^	5.93 ± 0.49^Bb^	4.63 ± 0.27^Aa^	6.54 ± 0.36^Ab^
	No. of filled seeds	Zero	82.90 ± 15.66^Aa^	252.96 ± 27.71^Bb^	311.18 ± 30.45^Bbc^	279.67 ± 14.44^Abc^	321.13 ± 27.16^Ac^
		Low	138.82 ± 25.02^Ba^	210.85 ± 24.32^ABb^	142.78 ± 16.24^Aa^	285.45 ± 21.49^Ac^	312.00 ± 26.03^Ac^
		Medium	88.02 ± 18.36^ABa^	232.43 ± 29.23^ABc^	149.32 ± 20.44^Ab^	229.65 ± 20.59^Ac^	476.64 ± 30.72^Bd^
		High	91.18 ± 20.76^ABa^	177.34 ± 19.00^Ab^	154.80 ± 17.26^Ab^	255.67 ± 20.72^Ac^	351.54 ± 20.35^Ad^
	Weight of filled seeds (g)	Zero	1.70 ± 0.34^Aa^	5.59 ± 0.62^Bb^	6.65 ± 0.72^Bbc^	6.68 ± 0.36^ABbc^	7.61 ± 0.66^Ac^
		Low	2.51 ± 0.46^ABa^	4.39 ± 0.46^ABb^	2.90 ± 0.34^Aa^	7.05 ± 0.54^Bc^	7.51 ± 0.67^Ac^
		Medium	1.60 ± 0.34^Aa^	5.24 ± 0.66^ABc^	3.02 ± 0.42^Ab^	5.58 ± 0.52^Ac^	11.00 ± 0.74^Bd^
		High	1.68 ± 0.38^Aa^	3.81 ± 0.42^Ab^	3.13 ± 0.36^Ab^	5.99 ± 0.49^Ac^	8.25 ± 0.58^Ad^
	100-seed weight (g)	Zero	1.81 ± 0.06^Ba^	2.15 ± 0.04^Ab^	2.14 ± 0.08^Bb^	2.39 ± 0.02^Ac^	2.35 ± 0.02^Ac^
		Low	1.64 ± 0.08^ABa^	2.03 ± 0.03^Ab^	1.95 ± 0.06^ABb^	2.46 ± 0.02^Bc^	2.34 ± 0.03^Ac^
		Medium	1.62 ± 0.08^ABa^	2.09 ± 0.08^Ab^	1.77 ± 0.10^Aa^	2.39 ± 0.02^Ac^	2.29 ± 0.02^Ac^
		High	1.51 ± 0.12^Aa^	2.02 ± 0.07^Ab^	1.99 ± 0.04^Bb^	2.33 ± 0.03^Ac^	2.33 ± 0.01^Ac^
	Ratio of effective tillers per plant	Zero	0.95 ± 0.02^Ba^	0.96 ± 0.01^Ba^	0.98 ± 0.01^Cab^	1.00 ± 0.00^Cb^	0.98 ± 0.01^Aab^
		Low	0.72 ± 0.04^Aa^	0.95 ± 0.01^Bc^	0.90 ± 0.02^Bb^	0.97 ± 0.01^BCc^	0.97 ± 0.01^Ac^
		Medium	0.70 ± 0.04^Aa^	0.93 ± 0.02^Bb^	0.77 ± 0.04^Aa^	0.95 ± 0.01^Bb^	0.98 ± 0.01^Ac^
		High	0.70 ± 0.05^Aa^	0.87 ± 0.02^Ac^	0.79 ± 0.03^Ab^	0.90 ± 0.02^Acd^	0.94 ± 0.02^Ad^

*Different letters following the average values in the same rows indicate significant differences in levels of fitness-relative traits under four insect pressures according to Duncan's multiple range test. The capital letters represent the comparison of the same population among four insect pressures, and the lower-case letters represent the comparison among five populations under the same insect pressure*.

Under insect pressure, the performance of KF8 was significantly better than that of MH86. Except for the increased number of filled seeds under medium pressure, the other traits of the KF8 population did not significantly change along the insect pressure gradient, whereas the performance of MH86 decreased with the increase in insect pressure. At low and medium insect pressures, TP had more filled seeds and a much higher filled seed weight than TN, with increases of 48–56% and 18–51%, respectively. Under the same pressure, TN performed better than W in plant height, numbers of effective tillers and filled seeds, and 100-seed weight. Although the performance of the W population decreased with increasing insect pressure under existing insect pressure, it was equal or slightly higher than that under no insect pressure. The W populations had the highest values of effective tiller and filled seed numbers under low pressure. The TN population underperformed under insect pressure compared with those not under insect pressure. The TP population also had a 20–27% reduction in aboveground biomass under low and medium insect pressure and a smaller aboveground biomass and seed number under high insect pressure compared to the no insect pressure treatment (Table 2).

### Variation in Relative Fitness

**Figure 2:**
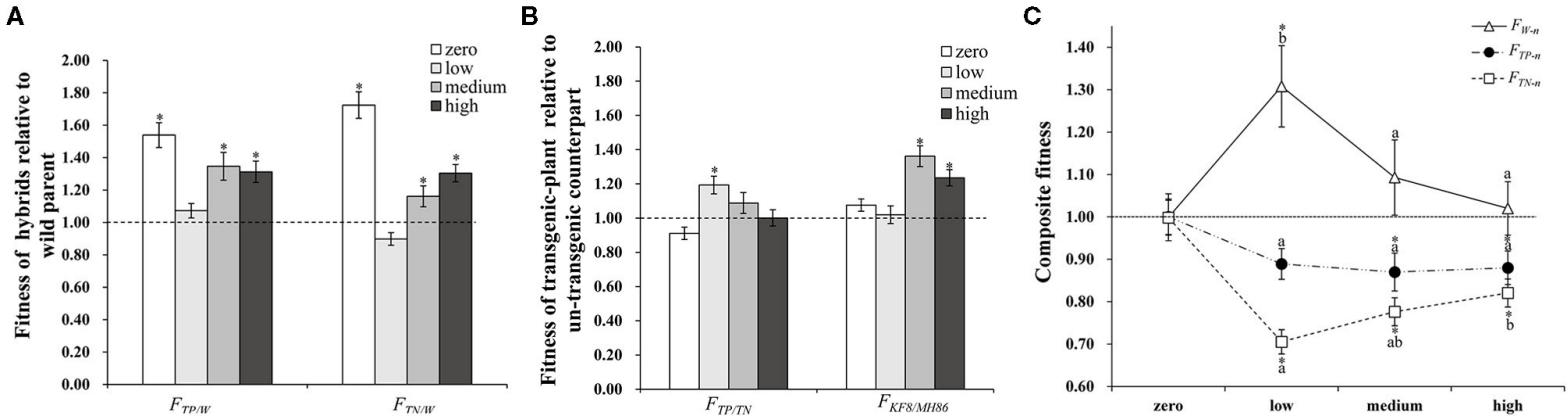
**(A)** The composite fitness of TP and TN relative to W (*F*_*TP*/*W*_ and *F*_*TN*/*W*_). **(B)** The composite fitness of transgenic plant relative to its non-transgenic counterpart (*F*_*TP*/*TN*_ and *F*_*KF*8/*MH*86_). **(C)** The composite fitness of transgenic and non-transgenic hybrids (TP and TN) and the wild parent (W) under insect pressure relative to conditions without insect pressure (*F*_*W*−*n*_, *F*_*TP*−*n*_, *F*_*TN*−*n*_), different letters denote their significant differences (*P* < 0.05) under three insect pressures using the Duncan's multiple range test. Bars represent standard error. Asterisk indicates the fitness value is significantly different from 1.00 based on the *t*-test (*P* < 0.05).

Plant fitness at each life stage varied between plant populations and was influenced by the interaction between population and insect pressure. In addition, the fitness parameters associated with growth and sexual reproduction were significantly influenced by insect pressure (Table 3). The changes in insect pressure significantly influenced the growth of the TP, TN, and MH86 populations, the clonal reproduction of the W, TN, and KF8 populations, the sexual reproduction of the TN and KF8 populations, and the composite fitness of the W, TN, KF8, and MH86 populations (Supplementary Table 3). In particular, the clonal reproduction of the W populations had 53, 40, and 30% increases while that of the hybrid populations decreased under low, medium, and high insect pressure, respectively, indicating over-compensation in wild plants and under-compensation in hybrids. The *t*-test revealed that *F*_*TN*/*W*_ and *F*_*TP*/*W*_ were significantly >1.00 (1.72 and 1.54) under no insect pressure, but when insect pressures occur the two values dramatically decreased, and the biggest reduction occurred under low insect pressure. Under the same level of insect pressure, *F*_*TP*/*W*_ was slightly higher than *F*_*TN*/*W*_ (Figure 2A; Supplementary Table 4). *F*_*TP*/*TN*_ was significantly >1.00 only under low pressure, whereas *F*_*KF*/*MH*86_ was significantly >1.00 under medium and high pressure (Figure 2B; Supplementary Table 4). *F*_*TN*/*W*_ and *F*_*TP*/*W*_ or *F*_*KF*/*MH*86_ and *F*_*TP*/*TN*_ had similar variation tendencies with the increase in the degree of insect pressure (χ^2^, *p* > 0.05) (Supplementary Table 5). Fitness comparisons between populations under insect pressure and those not under insect pressure showed that, for the W population, the fitness related to clonal reproduction and the composite fitness both showed *F*_*W*−*l*_ > *F*_*W*−*m*_ > 1.00 and *F*_*W*−*h*_ = 1.00 (Figure 2C; Supplementary Table 6); for the populations TP and TN, *F*_*TP*−*l*, −*m*, −*h*_ and *F*_*TN*−*l*, −*m*, −*h*_ were < 1.00 (Figure 2C; Supplementary Table 6); for the population MH86, the fitness related to clonal reproduction showed *F*_*MH*86−*l*_ > 1.00 and the other *F*_*MH*86−*n*_ = or <1.00 (Supplementary Table 6); for the KF8 population, several fitness estimates (*F*_*KF*8−*n*_) were >1.00 (Supplementary Table 6).

**Table 3:** *F*-values generated from two-way ANOVA testing the effects of rice type, insect pressure levels, and their interaction on fitness at different life-history stages and across the lifetime.

**Source**	***df***	**Relative fitness**			
		**Composite**	**Growth**	**Clonal reproduction**	**Sexual reproduction**
Rice type	4	22.722^**^	15.129^**^	25.391^**^	11.946^**^
Pressure	3	1.187	7.441^**^	2.550	2.936^*^
Rice type × Pressure	12	5.053^**^	2.927^**^	5.11^**^	3.478^**^

* *P < 0.05*;

** *P < 0.01*.

## Discussion

### Hybrid Vigour and Crop-Wild Hybrids Fitness

The comparisons between hybrids and wild plants revealed that the hybrids had a taller plant height, much greater aboveground biomass, tillers, and seed yields, consequently causing a higher relative fitness in the hybrids (*F*_*TN*/*W*_) (Table 2; Supplementary Table 4) and indicating a hybrid vigour. Similar to the findings of Burke et al. (1998) in irises, the present study also found that the hybrids exhibited the high clonal reproduction of the wild parent and the high seed production of the cultivated parent and thus performed better than the parents. This demonstrates that the eminent recombination of the parent traits may improve the fitness of the hybrid progeny (Mercer et al., 2007; Rose et al., 2009). A similar pattern was also detected in sunflower (Mercer et al., 2007), lettuce (Hartman et al., 2012), radish (Hegde et al., 2006, Snow et al., 2009), and rape (Rose et al., 2009). Together, these findings suggest that some domestication traits and genome segments originating from crops may maintain and enhance crop-wild hybrid fitness (Mercer et al., 2007; Corbi et al., 2017; Presotto et al., 2019), although crop traits are expected to reduce fitness in wild populations (Stewart et al., 2003; Hails and Morley, 2005).

Hybrid vigour is usually detected in early-generation hybrids (such as F1 hybrid) (e.g., Li et al., 2016) and is segregated in subsequent generations (Rhode and Cruzan, 2005; Hooftman et al., 2008). While this study detected hybrid vigour by recombination of the relative high levels of traits of parents in the F7 hybrids (Table 2; Supplementary Table 4). Other researchers also found a hybrid vigour in advanced crop-weed/wild hybrids (e.g., Campbell et al., 2006; Yang et al., 2012). Transgressive segregation, due to the combinations of positively contributing alleles, may play a critical role in the hybrid vigour of late-generation hybrids (Hartman et al., 2012). This point was also mentioned in a recent study (Wang et al., 2017). Hooftman et al. (2008) compared the hybrids produced by the autogamous and backcross pathways and showed that the hybrid vigour decreased with generations faster in the backcross hybrids. This study could know whether hybrid vigour also exists in advanced backcross progenies since the absence of backcross controls. Further study combining morphological and molecular measurements will help to reveal the mechanism underlying the hybrid vigour in advanced hybrid progeny.

### Fitness Effect of Transgenes

Transgenes are expected to have selection advantages under targeted environmental pressure. A number of studies support this expectation (e.g., Mercer et al., 2007; Londo et al., 2011; Xia et al., 2011, 2016; Yang et al., 2011, 2012, 2017; Li et al., 2016; Supplementary Figure 1). The trial used in this study involving four levels of insect pressure clearly revealed the fitness effects of the transgene. Under the condition of no insect pressure, the expression of the *Bt/CpTI* transgene in hybrids caused somewhat decreased fitness, i.e., a slight costly effect, as in the results of Yang et al. (2011) and Xia et al. (2016). When insect pressure occurred, the *Bt/CpTI* transgene effectively protected the rice plants from insect damage, and a lower insect density occurred in the transgenic population TP and KF8. Even the rice stem borer *C. suppressalis* did not survive to the next generation in the TP population at low and medium levels of initial insect infestation (Figure 1A), indicating the transgene's beneficial effects (Xia et al., 2011, 2016; Yang et al., 2011, 2012; Li et al., 2016). Given this case, the transgenic hybrid populations should have corresponding higher levels of fitness. However, the relative fitness of TP (*F*_*TP*/*TN*_) increased only under low levels of insect pressure and decreased with increasing insect pressure (from 1.19 to 1.00) (Figure 2B; Supplementary Table 4). This finding demonstrates that the transgene's effect on fitness is condition-dependent (Campbell et al., 2006; Mercer et al., 2007; Londo et al., 2011; Yang et al., 2011, 2012). Moreover, the present study showed that the transgenic and non-transgenic hybrids had the same trend in relative fitness with regard to increasing insect pressure (χ^2^, *p* > 0.05) (Supplementary Table 5). This result demonstrates the limited effects of the *Bt/CpTI* transgene on the fitness landscape of crop-wild hybrid progenies when a hybrid vigour exists. The transgenic cultivated rice had an inconsistent pattern of fitness under different insect pressures compared to the transgenic crop-wild rice hybrids (Supplementary Table 5). Genetic differences between the two rice plants accounted for their different degrees of fitness (Mercer et al., 2007; Xia et al., 2016; Dong et al., 2017).

### Compensation of Wild Parent and Relative Fitness of Hybrid

**Figure 3:**
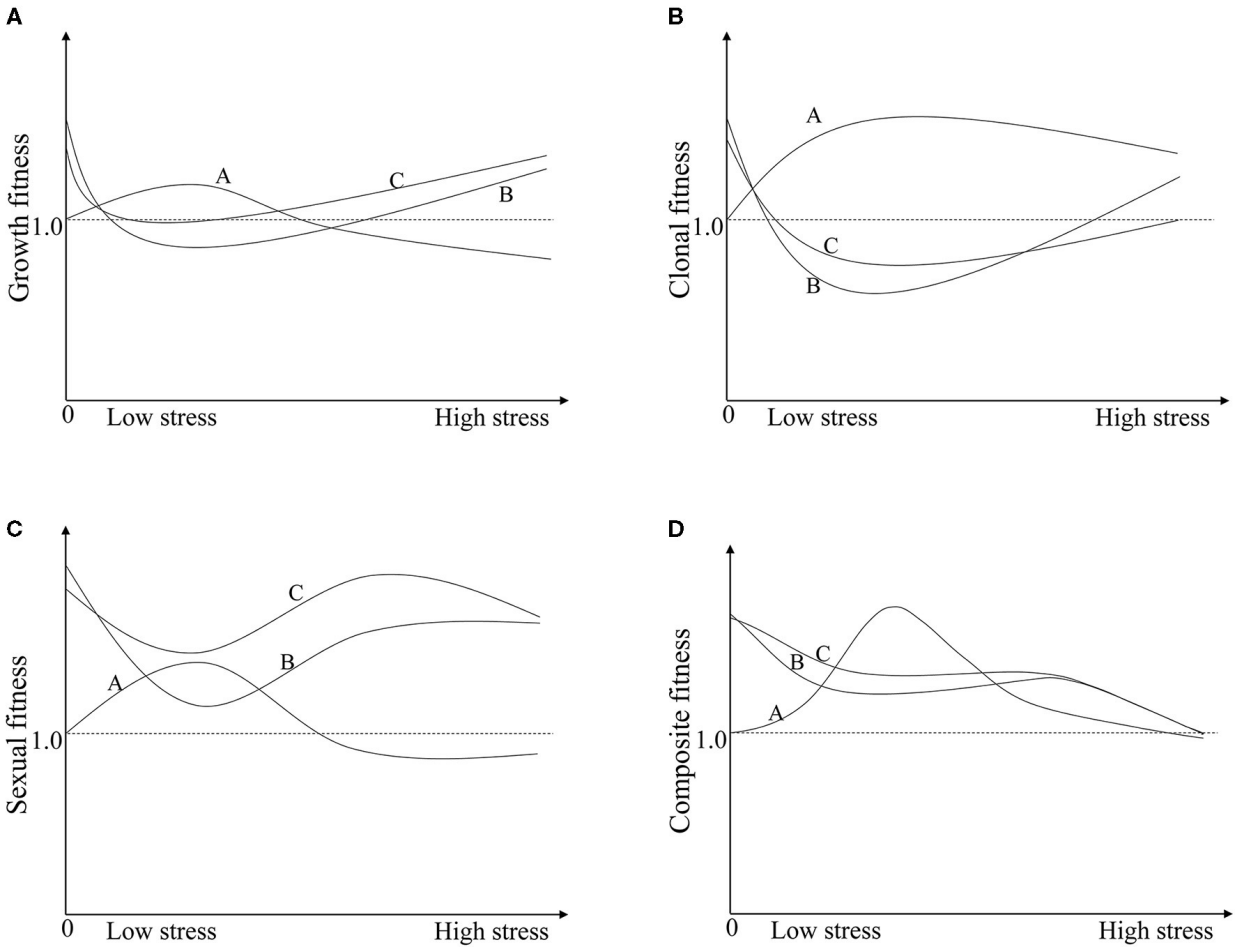
The schematic diagram illustrates the variations in fitness between crop-wild hybrids (TN and TP) and the wild parent (W) along pressure gradients. Line A, W; Line B, TN; Line C, TP. **(A)** The growth fitness of W, TN, and TP; **(B)** The clonal fitness of W, TN, and TP; **(C)** The sexual fitness of W, TN, and TP; **(D)** The composite fitness of W, TN, and TP. In graph **(D)**: Line A, wild plants are able to equally- or overcompensate for insect pressure to a certain degree, then fitness increases under moderate insect pressure and decreases, eventually becoming negative, at a higher insect pressure. Line B, non-transgenic crop-wild hybrids under-compensate for insect pressure but display a hybrid vigour; then, the fitness decreases with the insect pressure. Line C, the fitness of transgenic crop-wild hybrids increases because of the transgene relative compared to that of their non-transgenic counterparts under certain pressure conditions. The cross points between lines A and B or C indicate that the effects of hybrid vigour or hybrid vigour and transgenes are fully offset by compensation.

The hybrids have different compensatory responses to insect damage than their wild parents, showing different insect-tolerance. One interpretation of compensation is that the damage of herbivores may trigger the activation of basal or dormant meristems, inducing regrowth of lateral shoots to increase branching or tillering (Belsky et al., 1993; Strauss and Agrawal, 1999; Fornoni, 2011). For instance, Lv et al. (2008) found that rice stem injury could be compensated by additional tillers. Similarly, in the present study, wild plants had 19–35% more tillers per plant under insect pressure relative to under no insect pressure, resulting in a 30–53% increase of clonal reproduction fitness and 6–67% more seeds (Table 2; Supplementary Table 6; Figure 3B). Consequently, 2–31% increased composite fitness was detected in wild plants (Table 2; Figure 2C), indicating equal or over-compensation effects (Belsky, 1986; Schimmel et al., 2017), i.e., relative higher insect-tolerance. The cultivated rice MH86, which may be the maternal parent of the non-transgenic hybrid TN, exhibited equal- or under-compensation due to the similarly clonal reproduction fitness (Supplementary Table 6), whereas TN displayed under-compensation under insect pressure due to the slightly low clonal reproduction fitness and significantly lower growth and sexual reproduction fitness (Figures 2C, 3A,C), showing lower insect-tolerance. The different insect-tolerance between hybrids and parents can mainly be attributed to their different genetic backgrounds, which determine the plant's resource allocation and response to environmental variations (McNaughton, 1983; Lennartsson et al., 2018).

The compensation difference markedly influenced the hybrids' fitness relative to that of wild plants, resulting in decreased hybrid fitness under the insect existing conditions (Figure 2C), and its relative value was further decreased by the increased fitness of the wild plants (Figure 2A). For instance, the fitness of the TN hybrid relative to that of wild rice (*F*_*TN*/*W*_) increased by 72% under no insect pressure due to hybrid vigour, but the increasing rate of fitness decreased to −8, 16, and 30% under low, medium and high levels of insect pressure. While under low, medium and high levels of insect pressure, the fitness values of W (*F*_*W*−*n*_) increased with the rate of 31, 9, 2%. A similar pattern occurred in the transgenic hybrid TP, although the relative fitness (*F*_*TN*/*W*_) was increased both by transgenes and hybrid vigour (Figure 2B; Supplementary Table 4). The compensation difference can be further illustrated by a schematic diagram of the variations in fitness between crop-wild hybrids and the wild parent along pressure gradients (Figure 3). Figure 3 shows that the fitness values of the TN hybrid (Line B) seem slightly lower than those of the TP hybrid (Line C) and the two hybrids have the similar changing patterns of growth, clonal, sexual, and composite fitness components, which remarkably differ from those of wild parent (Line A), especially for the clonal fitness (Figure 3B). This means that wild rice can compensate the insect damage by increased tillering. More tillers may lead to additional reproductive tillers (Lv et al., 2008), resulting in the higher sexual and composite fitness of wild parents under most insect pressures (Figures 3C,D). These findings indicate that the effects of hybrid vigour and transgenes on fitness, though they exist, are both weakened by compensation (Mercer et al., 2007); and once again suggest the importance of clonal reproduction via tillers and stolons for the survival of wild rice *O. rufipogon*. Higher compensation means higher tolerance to insect damage. High tolerance (resistance) of wild rice was formerly suggested by Li et al. (2016) although there was no significant difference in insect damages between wild rice and transgene crop-wild hybrid descendants under high insect pressure. In addition, the compensatory effects on plant fitness in the current study varied with the degree of insect pressure (Figure 2C), as in the findings of Huhta et al. (2003). All of this again emphasising the importance of the pressure levels for fitness estimation.

### Influential Factors to Hybrid Fitness Performance

The present study shows that transgenic hybrid fitness is influenced not only by the transgenes *per se* but also by other factors, such as hybrid vigour and compensation. Consequently, the transgenic hybrids did not always perform significantly better than non-transgenic hybrids or their wild parents in most insect infestation scenarios (Figure 2B). These contributing factors included transgenes, hybrid vigour, compensation, and environmental conditions. The effects of each factor and their interactions are briefly summarised in Figure 3D and are as follows: (1) Hybrid vigour, if it exists (31.8% of observations showed hybrid vigour in our literature search, Supplementary Figure 1), improves hybrid fitness, which is an independent environmental condition (see line B in Figure 3D); (2) Nearly 32% of observations showed that the selective advantage of transgenes enhanced hybrid performance (Supplementary Figure 1). Such effects will add to that of hybrid vigour (see line C in Figure 3D) and are limited under certain environmental conditions; (3) Compensation effects of crop-wild hybrids and their parents both varied with the intensity of environmental pressure. If hybrids show under-compensation compared with their wild parent, e.g., under- and overcompensation in hybrids and wild rice here, respectively (Figure 2, line A in Figure 3D), then the hybrids have a lower composite fitness, suggesting a negative effect of compensation on hybrid vigour or/and transgenes. Under extreme conditions, compensation could fully offset the additive effects of transgenes and hybrid vigour; (4) The interactions between transgenes, compensation, or/and hybrid vigour strongly depend on the growing environments, and their combined effects determine whether transgenic crop-wild hybrids perform better than non-transgenic hybrids.

### Implications for Crop-Wild Gene Flow

The present study reveals that the performance of transgenic hybrids is determined by the interactions between the effects of transgenes, hybrid vigour, and compensation. Hybrid vigour and transgenes may increase hybrid fitness together, but compensation reduces hybrid fitness under insect stressful environments. In addition to herbivore damage, natural populations often suffer from other stresses, such as pathogen infection, chilling, drought, and/or competition, to which wild plants generally display stronger tolerance than crops. For instance, wild sunflower populations have greater tolerance to the competition of other plants or to herbicide damage than hybrids (Mercer et al., 2007). The selective advantage of transgenes disappears in the absence of a targeted pressure, but tolerance might still occur if other stresses exist. Certainly, the high compensation of wild parent will decrease the opportunity for crop-wild hybrid survival in the natural environment. Compensation is therefore a mechanism that may not eliminate but can at least weaken the impacts of crop-wild gene flow on wild plants.

Tolerance and resistance traits are both mechanisms of plant defence against herbivores that confer a fitness benefit to the plant in the presence of herbivores (Strauss and Agrawal, 1999; Agrawal, 2000). The existing insect-tolerance of wild populations could result in an equally or even better variable relative fitness of crop-wild hybrids carrying an insect-resistance transgene (Mercer et al., 2007). Natural selection stemming from wild conditions overwhelmingly favours wild alleles and phenotypes rather than crop genes or traits, even those which can provide natural selective advantages (Corbi et al., 2017). Therefore, the wild populations could keep their “wild” characteristics. Here, the joint effects of selective advantages of crop alleles (transgenes) and hybrid vigour on a plant's performance is weakened by compensation. This pattern can be attributed to the compensation effect of wild parents. The effects of other crop alleles (e.g., *sh4* and *PROG1*) on the performance of wild rice can also be compensated (Wang et al., 2017). Mercer et al. (2007) also showed that the susceptibility to herbicide damage could be compensated by relatively larger seedlings in wild sunflower populations. High tolerance of wild rice *O. rufipogon* mainly expressed by the compensated clonal growth (Figure 3B), which has implications for maintaining the perennial habit. Given the case of compensation effect, the extant wild population can maintain the “wild” characteristics while the crop alleles, whether advantageous or disadvantageous in a variety of natural environments, persist.

In addition, the domestication traits assumed to be detrimental or beneficial may, in practical terms, hardly affect wild populations. We do not claim that the fitness effect of all crop alleles could be compensated by the tolerance of wild plants under any stress environments. The present study reveals that the interactions between genetic and environmental factors are extraordinarily complicated (Figure 3). It calls for a more comprehensive environmental stress setting design for future studies of crop-wild hybrid fitness. Such design will provide a more accurate estimates of the ecological impacts of crop gene introgression. Moreover, our study confirms that the response of relative crop-wild hybrid fitness to a given stress depends on the tendency of crop traits to confer tolerance or susceptibility and phenotypic traits of the wild plant to the stress, the gene action of the crop alleles (transgenes) involved. Thus, much attention should be payed to the phenotypic traits with similar functions to domestication traits in wild populations in evaluating the risk of transgene escaping.

## Data Availability Statement

The original contributions presented in the study are included in the article/Supplementary Material, further inquiries can be directed to the corresponding author/s.

## Author Contributions

ZS, YW, WZ, and JYa conceived the study. DO, SD, MX, JYo, and YZ collected the data. DO performed the statistical analyses, wrote the first draught of manuscript, and all authors contributed substantially to revisions.

## Conflict of Interest

The authors declare that the research was conducted in the absence of any commercial or financial relationships that could be construed as a potential conflict of interest.
