# Supplementary material for: Compensation of Wild Plants Weakens the Effects of Crop-Wild Gene Flow on Wild Rice Populations
Source: Front Plant Sci. 2021 Jul 13;12:681008. doi: 10.3389/fpls.2021.681008 (PMC8314011; doi:10.3389/fpls.2021.681008)
Supplement: Supplementary file 1 [file Data_Sheet_1.docx]

**Compensation of wild plants weakens the effects of crop-wild gene flow on wild rice populations**

**TABLE S1.** Traits used to estimate life-history composite fitness.

| **Life-history stage** | **Trait** | **Method of measurement** |  |
| --- | --- | --- | --- |
| Growth | Plant height (cm) | Height from the base of plant (soil line) to the tip of panicles of the main tillers at maturity |  |
|  | Aboveground biomass (g) | Drying and weighing of each plant after harvest |  |
|  | Growth investment (g) | Drying and then removing filled seeds and weighing each plant after harvest |  |
| Reproductive | No. of tillers | Number of tillers determined at maturity for each plant |  |
|  | No. of filled seeds | Drying and weighing of each plant after harvest |  |
|  | No. of panicles | Number of panicles per plant at maturity |  |
|  | No. of filled seeds per panicles | Ratio of filled seeds and number of panicles | |
|  | Weight of filled seeds (g) | Drying and weighing of each plant after harvest |  |
|  | 100-seeds weight (g) | Drying and weighing of each plant after harvest |  |
|  | Ratio of productive tillers | Ratio of panicles and tillers for each plant at maturity |  |

**TABLE S2.** Comparison of the final and the initial population densities of *C. suppressalis* in each type of plant population was done using the chi-square test. Significant *P*-values (P ˂ 0.05) are indicated with bold type.

| Rice type | χ^2^ value | *df* | *P*-value |
| --- | --- | --- | --- |
|  |  |  |  |
| W | 23.848 | 2 | **0.000** |
| TP | 13.273 | 2 | **0.001** |
| TN | 48.342 | 2 | **0.000** |
| MH86 | 64.484 | 2 | **0.006** |
| KF8 | 1.014 | 2 | 0.602 |

**TABLE S3.** *F*-values generated from ANOVA analysis of the effects of insect pressure on relative fitness at different life-history stages and on composite fitness.

| Relative fitness | *df* | *F_W-n_* | *F_TP-n_* | *F_TN-n_* | *F_KF8-n_* | *F_MH86-n_* |  | *F_TP/W_* | *F_TN/W_* |  | *F_TP/TN_* | *F_KF8/MH86_* |
| --- | --- | --- | --- | --- | --- | --- | --- | --- | --- | --- | --- | --- |
| Growth | 3 | 2.186 | 4.160** | 8.528** | 0.611 | 7.854** |  | 4.268** | 11.593** |  | 2.163 | 11.641** |
| Clonal reproduction | 3 | 6.083** | 1.589 | 7.896** | 3.582* | 1.319 |  | 19.388** | 31.17** |  | 7.312** | 5.298** |
| Sexual reproduction | 3 | 1.883 | 1.769 | 14.701** | 6.868** | 2.52 |  | 4.918** | 17.235** |  | 6.94** | 13.982** |
| Composite | 3 | 3.402* | 2.219 | 13.362** | 3.321* | 3.125* |  | 7.505** | 30.794** |  | 5.806** | 12.441** |

*F_W-n_*, *F_TP-n_*, *F_TN-n_*, *F_KF8-n_*, *F_MH86-n_* the fitness of population W, TP, TN, KF8 and MH86 under insect pressures relative to levels under no insect pressure, respectively. ***F*** *_TP/W_*, ***F*** *_TN/W_*, the fitness of transgenic and non-transgenic hybrids relative to the wild parent under 4 levels of insect pressure, respectively. ***F*** *_TP/TM_*, ***F*** *_KF/MH86_* the fitness of the transgenic hybrid and transgenic parent relative to their non-transgenic counterparts under 4 levels of insect pressure, respectively.

* *P*＜0.05; ** *P* ＜0.01.

**TABLE S4.** Average values and standard errors (±) of the fitness of different life-history stages of transgenic-positive (TP) and transgenic-negative (TN) crop-wild hybrid progeny relative to the wild rice parent (W), transgenic-positive hybrid (TP) relative to transgenic-negative hybrid (TN), transgenic-positive cultivated rice (KF8) relative to transgenic-negative cultivated rice (MH86) under 4 insect pressures.

| Relative fitness | Growth | Clonal reproduction | Sexual reproduction |
| --- | --- | --- | --- |
| *F_TP/W_* |  |  |  |
| zero | 1.27±0.05** | 1.37±0.07** | 1.98±0.15** |
| low | 0.99±0.04 | 0.82±0.05* | 1.40±0.08** |
| medium | 1.13±0.08 | 0.86±0.05 | 2.06±0.18** |
| high | 1.27±0.07** | 1.02±0.05 | 1.65±0.12** |
| *F_TN/W_* |  |  |  |
| zero | 1.39±0.08** | 1.49±0.08** | 2.29±0.16** |
| low | 0.88±0.04 | 0.71±0.04** | 1.10±0.06 |
| medium | 1.06±0.07 | 0.98±0.05 | 1.45±0.14* |
| high | 1.22±0.06** | 1.20±0.07* | 1.50±0.10** |
| *F_TP/TN_* |  |  |  |
| zero | 0.92±0.04 | 0.92±0.05 | 0.90±0.05 |
| low | 1.13±0.05 | 1.17±0.07* | 1.28±0.07** |
| medium | 1.05±0.08 | 0.87±0.05 | 1.34±0.10** |
| high | 1.05±0.06 | 0.86±0.05* | 1.10±0.07 |
| *F_KF8/MH86_* |  |  |  |
| zero | 0.97±0.05 | 1.20±0.07* | 1.06±0.05 |
| low | 1.06±0.04 | 0.98±0.05 | 1.02±0.04 |
| medium | 1.33±0.05** | 1.26±0.07** | 1.50±0.06** |
| high | 1.23±0.05** | 1.29±0.16** | 1.18±0.04** |

The level of significance was calculated based on the t-test between the fitness of TP with W, TN with W, TP with TN, and KF8 with MH86 under the same insect pressure.

* *P*-value ˂ 0.05. ** *P*-value ˂ 0.01.

**TABLE S5.** Chi-square test for the variations of relative fitness *F_TP/W_* *vs.* *F_TN/W_* and *F_TP/TN_* *vs.* *F_KF8/MH86_* along the insect pressure gradients.

|  | Insect pressure | | | | | | | χ^2^ value | *P*-value |
| --- | --- | --- | --- | --- | --- | --- | --- | --- | --- |
|  | zero | low | | medium | | high | |  |  |
| *F_TP/W_ / F_TN/W_* | 1.54/1.72 | | 1.07/0.90 | | 1.35/1.16 | | 1.31/1.30 | 3.555 | 0.314 |
| *F_TP/TN_ / F_KF8/H86_* | 0.91/1.08 | | 1.19/1.02 | | 1.09/1.36 | | 1.00/1.24 | 5.449 | 0.142 |

**TABLE S6.** Average values and standard errors (±) of fitness for different life-history stages of crop-wild hybrids and their parents under 3 insect pressures relative to fitness under 0 insect pressure conditions.

| Relative fitness | Growth | Clonal reproduction | Sexual reproduction |
| --- | --- | --- | --- |
| *F_W-n_* |  |  |  |
| low | 1.12 ±0.08^b^ | 1.53±0.12^a^** | 1.27±0.15^b^ |
| medium | 0.98±0.09^ab^ | 1.40±0.12^a^** | 0.90±0.11^a^ |
| high | 0.85±0.05^a^ | 1.30±0.08^a^* | 0.91±0.12^a^ |
| *F_TP-n_* |  |  |  |
| low | 0.86±0.04^a^* | 0.90±0.05^a^ | 0.90±0.04^ab^ |
| medium | 0.80±0.04^a^** | 0.85±0.05^a^* | 0.95±0.07^b^ |
| high | 0.85±0.04^d^* | 0.97±0.05^a^ | 0.83±0.05^a^* |
| *F_TN-n_* |  |  |  |
| low | 0.72±0.03^a^** | 0.71±0.04^a^** | 0.68±0.03^a^** |
| medium | 0.76±0.04^a^** | 0.89±0.04^b^ | 0.68±0.05^a^** |
| high | 0.74±0.03^a^** | 1.03±0.07^c^ | 0.70±0.04^a^** |
| *F_MH86-n_* |  |  |  |
| low | 0.96±0.04b | 1.16±0.07a* | 1.04±0.05b |
| medium | 0.79±0.04a** | 1.06±0.06a | 0.89±0.04a* |
| high | 0.86±0.03a** | 1.08±0.05a | 0.94±0.04a |
| *F_KF8-n_* |  |  |  |
| low | 1.05±0.04a | 0.94±0.04a | 0.99±0.05a |
| medium | 1.06±0.04a | 1.11±0.06b | 1.24±0.05b** |
| high | 1.08±0.04a | 1.16±0.05b* | 1.04±0.04a |

The level of significance was calculated based on the t-test between low insect pressure (6 larvae/m^2^) and 0, medium insect pressure (12 larvae/m^2^) and 0, or high insect pressure (24 larvae/m^2^) and 0, * *P*-value ˂ 0.05. ** *P*-value ˂ 0.01.

Different letters following the average values in the same rows indicate significant differences in fitness among the 3 levels of insect pressure according to Duncan’s multiple range tests after Bonferroni correction.

**(a)**


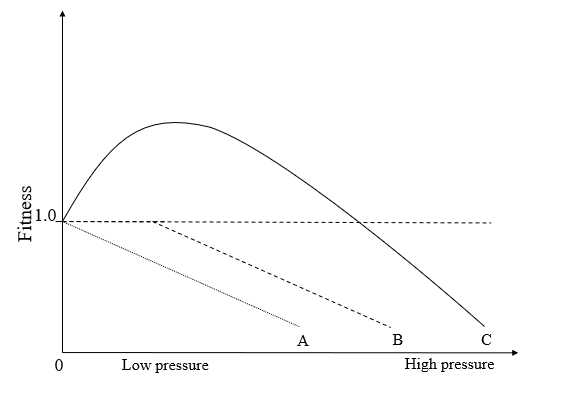
**(b)**

**Figure. S1. a.** A total of 328 observations reported in 39 studies (1998~2020) that focused on crop-wild/weed hybrid performance were used in this cursory literature review, transgenic vs. non-transgenic hybrids (n=107) (*F_TP/TN_*) and hybrids vs. wild/weed parents (n=221) (*F_H_* represents the fitness of non-transgenic and conventional hybrids relative to the wild parent, and *F_TP_* represents the fitness of transgenic hybrid relative to the wild parent), respectively. Among these and hybrids vs. wild/weed parent studies, 33.48% (n=74, 41 under environmental pressures) showed higher hybrid fitness, 33.03% (n=73, 43 under environmental pressures) had lower hybrid fitness, and 33.48% (n=74, 37 under environmental pressures) displayed similar fitness performance between wild parents and hybrids. For the observations with increased hybrid fitness, 28.51% (n=63) and 71.49% (n=158) did and did not involve transgenes, respectively. A total of 25.23% observations (n=27, including 23 observations under environmental pressures) found that the transgenic plants performed better than their non-transgenic counterparts, whereas 63.55% (n=41 under environmental pressures) and 12 observations (n=8 under environmental pressures) showed that the transgenic plants had similar or poorer performance than their non-transgenic counterparts. We used the seed trait of transgenic and non-transgenic counterpart plants to estimate their fitness. The fitness score of each plant was calculated as the mean of the seed production of the plant, dividing the seed production of non-transgenic plants in the controlled treatment so that the fitness of non-transgenic plants in the controlled treatment was always assigned as 1.00. **b.** Three alternative hypotheses about the fitness of plants under pressure. **A**. Plant fitness declines consistently as the select pressure increases, showing under-compensation. B. Plants exhibit equal compensation for the negative impact of selection pressure to some degree, and fitness declines with increasing pressure. C. Plants have higher fitness and fitness increases with the level of pressure and reaches maximum values under certain stress conditions, showing over-compensation, then declines and becomes negative at higher levels of pressure. After McNaughton. 1983; Strauss and Agrawal. 1999; Huhta *et al*. 2003; Lennartsson *et al*. 2018.

References: experimental studies included in meta-analyses.

| Species | Hybrid cross | Environmental stress | Fitness comparison | Observation number | Reference |
| --- | --- | --- | --- | --- | --- |
| Rice | F1, F3 | control, drought | transgenic hybrid *vs* wild parent | 6 | Nam *et al*. *Scientific Reports*, 2020, 10(1): 9319 |
|  | F1, F2 | insect | transgenic *vs* non-transgenic hybrids | 20 | Xia *et al*. *Evolutionary Applications*, 2016, 9(7): 847-856 |
|  | F2, F3 | herbicide, competition | transgenic *vs* non-transgenic hybrids | 48 | Yang *et al*. *Evolutionary Applicatuions*, 2011, 4(5): 672-684 |
|  | F4 | insect and competition | transgenic *vs* non-transgenic hybrids | 12 | Yang *et al*. *Plos One*, 2012, 7(7): e41220 |
|  | F1-F3 | no | transgenic *vs* non-transgenic hybrids | 20 | Yang *et al*. *Scientific Reports*, 2017, 7(1): 6834 |
|  | F1 | drought, natural insect, competition | hybrid *vs* wild parent, transgenic hybrid vs wild parent, transgenic vs non-transgenic hybrids | 8 | Yao *et al*. *Journal of Integrative Agriculture*, 2019, 18(12): 2793-2805 |
| Sunflower | G3 | natural conditions | transgenic *vs* non-transgenic hybrids | 6 | Corbi *et al*. *Molecular Ecology*, 2017, 27(1): 233-247. |
|  | F1 | pathogenic bacteria | transgenic hybrid *vs* wild parent | 3 | Burke and Rieseberg *Science*, 2003, 300: 1250 |
|  | F2 | no | crop-wild hybrid *vs* wild parents | 2 | Gutierrez *et al*. *Plant Biology*, 2011, 13(5): 821-830 |
|  | F1 | competition | crop-wild hybrid *vs* wild parents | 4 | Mercer *et al*. *Evolution*, 2006, 60(10): 2044-2055 |
|  | F1 | herbicide | transgenic *vs* non-transgenic hybrids | 10 | Mercer *et al*. *Ecology Letters*, 2007, 10(5): 383-393 |
|  | BCw, F1, F2 | competition | crop-wild hybrid *vs* wild parents | 18 | Mercer *et al*. *Plos One*, 2014, 9(10): e109001 |
|  | F1, F5-F8 | no | crop-wild hybrid *vs* wild parents | 7 | Mitchell *et al*. *Scientific Reports*, 2019, 9(1): 6746 |
|  | F1 | leaf defoliation, drought | crop-wild hybrid *vs* wild parents | 4 | Presotto *et al*. *Agriculture Ecosystems & Environment*, 2017, 249: 12-21 |
|  | BC1 | competition | transgenic *vs* non-transgenic hybrids | 8 | Snow *et al*. *Ecological Applications*, 2003, 13(2): 279-286 |
| Radish | F1 | competition | crop-wild hybrid *vs* wild parents | 3 | Campbell *et al*. *Ecology Letters*, 2006, 9: 1198-1209 |
|  | F3 | competition | crop-wild hybrid *vs* wild parents | 6 | Campbell and Snow *New Phytologist*, 2007, 173(3): 648-660. |
|  | F1 | no | crop-wild hybrid *vs* wild parents | 2 | Campbell *et al*. *Canadian Journal of Plant Science*, 2014, 94(8): 1315-1324 |
|  | F5 | no | crop-wild hybrid *vs* wild parents | 3 | Campbell *et al*. *Evolutionary Applications*. 2016, 9(5): 697-708 |
|  | F1 | competition | crop-wild hybrid *vs* wild parents | 2 | Hovick *et al*. *The American Naturalist*, 2012, 179: 192-203 |
|  | Hybrid | no | crop-wild hybrid *vs* wild parents | 2 | Ridley and Ellstrand *Biological Invasions*, 2009, 11(10): 2251-2264 |
|  | F2 | competition | crop-wild hybrid *vs* wild parents | 2 | Snow *et al*. *Ecological Applications*, 2001, 11(3): 934-943 |
|  | F10 | competition | crop-wild hybrid *vs* wild parents | 3 | Snow *et al*. *New Phytologist*, 2010, 186: 537-548 |
| Rape | F1-BC2F2 | insect | transgenic hybrid *vs* wild parent | 6 | Halfhill *et al*. *Molecular Ecology*, 2005, 14(10): 3177-3189 |
|  | F1 | no | crop-wild hybrid *vs* wild parents | 2 | Hauser *et al*. *Heredity*, 1998, 81(4): 429-435 |
|  | F2 | no | crop-wild hybrid *vs* wild parents | 5 | Hauser *et al*. *Heredity*, 1998, 81(4): 436-443 |
|  | F2 | herbicide, competition | transgenic hybrid *vs* wild parent | 21 | Londo *et al*. *Heredity*, 2011, 107(4): 328-337 |
|  | BC2 | control, insect | transgenic *vs* non-transgenic hybrids | 8 | Liu *et al*. *Transgenic Research*, 2015, 24(3): 537-547 |
|  | BC1, F2 | competition | transgenic hybrid *vs* wild parent | 6 | Rose *et al*. *BMC Biotechnology*, 2009, 9(1): 93-107 |
|  | F1 | herbivory | hybrid *vs* wild parent, transgenic hybrid vs wild parent, transgenic vs non-transgenic hybrids | 12 | Sagers *et al*. *Agronomy*, 2015, 5(1): 21-34 |
|  | BC3 | no | transgenic hybrid *vs* wild parent | 18 | Snow *et al*. *Molecular Ecology*, 1999, 8(4): 605-615 |
| Lettuce | F1-BC3 | simulated natural | crop-wild hybrid *vs* wild parents | 8 | Hofftman *et al*. *Journal of Applied Ecology*, 2007, 44(4): 1035-1045 |
|  | RIL, BC1 | no | crop-wild hybrid *vs* wild parents | 4 | Hartman *et al*. *Evolutionary Applications*, 2013, 6(4): 569-584 |
|  | RIL | low nutrients, competition, drought, salt stress | crop-wild hybrid *vs* wild parents | 13 | Hartman *et al*. *Ecology and Evolution,* 2014, 4(12): 2395-2409 |
|  | F1 | no | crop-wild hybrid *vs* wild parents | 4 | Hooftman *et al*. *Environmental Biosafety Research*, 2009, 8(4): 203-217 |
|  | BC1S1, BC2S1 | no | crop-wild hybrid *vs* wild parents | 10 | Uwimana *et al*. *BMC Plant Biology*. 12(1): 43 |
| Maize | F1-F3 | no | transgenic hybrid *vs* wild parent | 3 | Guadagunolo and Ellstrand *Ecological Applications*, 2006, 16(5): 1967-1974 |
| Sorghums | F1 | competition | crop-wild hybrid *vs* wild parents | 8 | Magomere *et al*. *Journal of Biological Sciences*, 2015, 1727-3048 |
| Sugarcane | F1 | no | crop-wild hybrid *vs* wild parents | 1 | Pachakkil *et al*. *Scientific Reports*, 2019, 9(1): 1748 |

Burke, J.M. and Rieseberg, L.H. (2003). Fitness effects of transgenic disease resistance in sunflowers. *Science*. 300, 1250-1250.

Campbell, L.G. and Snow, A.A. (2007). Competition alters life history and increases the relative fecundity of crop-wild radish hybrids (*Raphanus spp*.). *New Phytologist*. 173, 648-660.

Campbell, L.G., Snow, A.A. and Ridley, C.E. (2006). Weed evolution after cultivated gene introgression: greater survival and fecundity of hybrids in a new environment. *Ecology Letters*. 9, 1198-1209.

Campbell, L.G., Teitel, Z. and Miriti, M.N. (2016). Contemporary evolution and the dynamics of invasion in crop-wild hybrids with heritable variation for two weedy life histories. *Evolutionary Applications*. 9, 697-708.

Campbell, L.G., Teitel, Z., Miriti, M.N. and Snow, A.A. (2014). Context-specific enhanced invasiveness of *Raphanus* crop-wild hybrids: A test for associations between greater fecundity and population growth. *Canadian Journal of Plant Science*. 94, 1315-1324.

Corbi, J., Baack, E.J., Dechaine, J.M., Seiler, G. and Burke, J.M. (2017). Genome-wide analysis of allele frequency change in sunflower crop-wild hybrid populations evolving under natural conditions. *Molecular Ecology*. 27, 233-247.

Guadagunolo, R. and Ellstrand, N. (2006). Relative fitness of transgenic vs. non-transgenic MAIZE x TROSINTE hybrids: a field evaluation. *Ecological Applications*. 16, 1967-1974.

Gutierrez, A., Cantamutto, M. and Poverene, M. (2011). Persistence of sunflower crop traits and fitness in *Helianthus petiolaris* populations. *Plant Biology*. 13, 821-830

Halfhill, M.D., Sutherland, J.P., Moon, H.S. *et al*. (2005). Growth, productivity, and competitiveness of introgressed weedy *Brassica rapa* hybrids selected for the presence of Bt *cry1Ac* and *gfp* transgenes. *Molecular Ecology*. 14, 3177-3189.

Hartman, Y., Hooftman, D.A.P., Uwimana, B. *et al*. (2014). Abiotic stress QTL in lettuce crop-wild hybrids: comparing greenhouse and field experiments. *Ecology and Evolution*. 4, 2395-2409.

Hartman, Y., Uwimana, B., Hooftman, D.A.P. *et al*. (2013). Genomic and environmental selection patterns in two distinct lettuce crop-wild hybrid crosses. *Evolutionary Applications*. 6, 569-584.

Hauser, T.P., Jørgensen, R.B. and Østergård, H. (1998). Fitness of backcross and F2 hybrids between weedy *Brassica rapa* and oilseed rape (*B. napus*). *Heredity*. 81, 436-443.

Hauser, T.P., Jørgensen, R.B. and østergård, H. (1998). Fitness of F1 hybrids between weedy *Brassica rapa* and oilseed rape (*B. napus*). *Heredity*. 81, 429-435.

Hooftman DAP., Hartman, Y., Oostermeijer, J.G.B. and den Nijs, J.C.M. (2009). Existence of vigorous lineages of crop-wild hybrids in Lettuce under field conditions. *Environmental Biosafety Research*. 8, 203-217.

Hooftman DAP., Jong, M.A.D., Oostermeijer, J.G.B. and den Nijs, J.C.M. (2007). Modelling the long-term consequences of crop-wild relative hybridization: a case study using four generations of hybrids. *Journal of Applied Ecology*. 44, 1035-1045.

Hovick, S.M., Campbell, L.G., Snow, A.A. and Whitney, K.D. (2012). Hybridization alters early life-history traits and increases plant colonization success in a novel region. *The American Naturalist*. 179, 192-203.

Liu, Y.B., Darmency, H., Neal, S.J.C., Wei, W., Tang, Z.X. and Ma, K.P. (2015). The effect of *Bt*-transgene introgression on plant growth and reproduction in wild *Brassica juncea*. *Transgenic Research*. 24, 537-547.

Londo, J.P., Bollman, M.A., Sagers, C.L., Lee, E.H. and Watrud L.S. (2011). Changes in fitness-associated traits due to the stacking of transgenic glyphosate resistance and insect resistance in *Brassica napus* L. *Heredity*. 107, 328-37.

Magomere, T.O., Obukosia, S.D., Shibairo, S.I. *et al*. (2015). Evaluation of relative competitive Ability and Fitness of *Sorghum bicolor×Sorghum halepense* and Sorghum bicolor×Sorghum sudanense F1 hybrids. *Journal of Biological Sciences*. 1727-3048.

Mercer K.L., Emry, D.J., Snow, A.A., Kost, M.A., Pace, B.A. and Alexander, H.M. (2014). Fitness of crop-wild hybrid sunflower under competitive conditions: implications for crop-to-wild introgression. *Plos One*. 9, e109001.

Mercer, K.L., Andow, D.A., Wyse, D.L. and Shaw, R.G. (2007). Stress and domestication traits increase the relative fitness of crop-wild hybrids in sunflower. *Ecology Letters*. 10, 383-393.

Mercer, K.L., Wyse, D.L. and Shaw, R.G. (2006). Effects of competition on the fitness of wild and crop-wild hybrid sunflower from a diversity of wild populations and crop lines. *Evolution*. 60, 2044-2055.

Mitchell, N., Owens, G.L., Hovick, S.M., Rieseberg, L.H. and Whitney, K.D. (2019). Hybridization speeds adaptive evolution in an eight-year field experiment. *Scientific Reports*. 9, 6746.

Nam K.H., Kim, D.Y., Moon, Y.S., Pack, I.S., Jeong, S.C., Kim, H.B. and Kim, C.G. (2020). Performance of hybrids between abiotic stress-tolerant transgenic rice and its weedy relatives under water-stressed conditions. *Scientific Reports*. 10, 9319.

Pachakkil, B., Terajima, Y., Ohmido, N., Ebina, M., Irei, S., Hayashi, H. and Takagi, H. (2019). Cytogenetic and agronomic characterization of intergeneric hybrids between *Saccharum* spp. hybrid and *Erianthus arundinaceus*. *Scientific Reports*. 9, 1748.

Presotto, A., Fernando, H., Marina, D. *et al*. (2017). Crop-wild sunflower hybridization can mediate weediness throughout growth-stress tolerance trade-offs. *Agriculture Ecosystems & Environment*. 249, 12-21.

Ridley, C.E. and Ellstrand, N.C. (2009). Evolution of enhanced reproduction in the hybrid-derived invasive, California wild radish (*Raphanus sativus*). *Biological Invasions*. 11, 2251-2264.

Rose, C.W., Millwood, R.J., Moon, H.S., Rao, M.R. and Stewart, C.N. (2009). Genetic load and transgenic mitigating genes in transgenic *Brassica rapa* (field mustard) × *Brassica napus* (oilseed rape) hybrid populations. *BMC Biotechnology*. 9, 93.

Sagers, C.L., Londo, J.P., Bautista, N., Lee, E.H., Watrud, L.S. and King, G. (2015). Benefits of Transgenic Insect Resistance in *Brassica* Hybrids under Selection. *Agronomy*. 5, 21-34.

Snow, A. A., Culley, T.M., Campbell, L.G., Sweeney, P.M., Hegde, S.G. and Ellstrand N.C. (2010). Long-term persistence of crop alleles in weedy populations of wild radish (*Raphanus raphanistrum*). *New Phytologist*. 186, 537-548.

Snow, A.A. and Culley, U.T.M. (2001). Fitness of hybrids between weedy and cultivated radish: implications for weed evolution. *Ecological Applications*. 11, 934-943.

Snow, A.A., Andersen, B. and Jorgensen, R.B. (1999). Costs of transgenic herbicide resistance introgressed from *Brassica napus* into weedy *B. rapa*. *Molecular Ecology*. 8, 605-615.

Snow, A.A., Pilson, D., Rieseberg, L.H., Paulsen, M.J., Pleskac, N. and Reagon, M.R. (2003). A *Bt* transgene reduces herbivory and enhances fecundity in wild sunflowers. *Ecological Applications*. 13, 279-286.

Uwimana, B., Smulders, M.J.M., Hooftman, D.A.P. *et al*. (2012). Crop to wild introgression in lettuce: following the fate of crop genome segments in backcross populations. *BMC Plant Biology*. 12, 43.

Xia H, Zhang H, Wang W. *et al*. (2016). Ambient insect pressure and recipient genotypes determine fecundity of transgenic crop-weed rice hybrid progeny: implications for environmental biosafety assessment: *Evolutionary Applications*. **9**, 847-856.

Yang X, Xia H, Wang W. *et al*. (2011). Transgenes for insect resistance reduce herbivory and enhance fecundity in advanced generations of crop-weed hybrids of rice. *Evolutionary Applications*. 4, 672-684.

Yang, X., Li, L., Jiang, X., Wang, W. and Lu, B.R. (2017). Genetically engineered rice endogenous 5-enolpyruvoylshikimate-3-phosphate synthase (*epsps*) transgene alters phenology and fitness of crop-wild hybrid offspring. *Scientific Reports*. 7, 6834.

Yang, X., Wang, F., Su, J. and Lu, B.R. (2012). Limited fitness advantages of crop-weed hybrid progeny containing insect-resistant transgenes (*Bt/CpTI*) in transgenic rice field*. Plos One*. 7, 398.

Yao, H., Wang, Y.Y., Qiang, S., Song, X.L. and Dai, W.M. (2019). Fitness of F1 hybrids between stacked transgenic rice T1c-19 with *cry1C*/bar* genes and weedy rice. *Journal of Integrative Agriculture*. 18, 2793-2805.

**
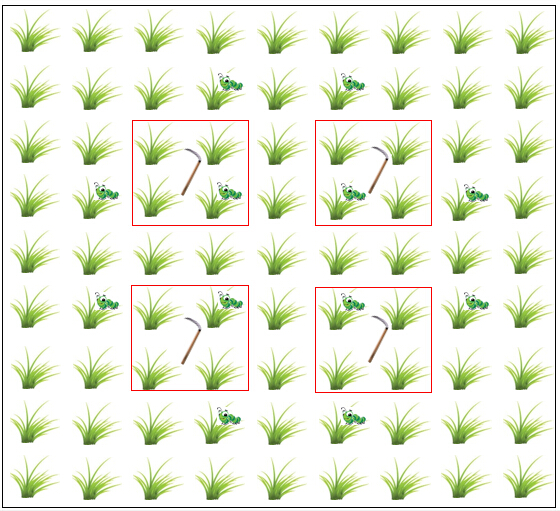
Figure. S2.** The insect site indicates the initial site of *C. suppressalis* larvae release*,* and the plants in the red boxes were harvested from each plot.
